# Cognitive Performance in Patients With Alcohol-Associated Liver Disease Undergoing Liver Transplantation

**DOI:** 10.3389/ti.2025.12869

**Published:** 2025-09-05

**Authors:** Magdalena Grusiecka-Stańczyk, Maciej K. Janik, Piotr Olejnik, Aleksandra Golenia, Jolanta MaƗyszko, Joanna Raszeja-Wyszomirska

**Affiliations:** ^1^ Department of Hepatology, Transplantology and Internal Medicine, Medical University of Warsaw, Warsaw, Poland; ^2^ Medical University of Warsaw, Warsaw, Poland; ^3^ Department of Neurology, Medical University of Warsaw, Warsaw, Poland; ^4^ Department of Nephrology, Dialysis, and Internal Medicine, Medical University of Warsaw, Warsaw, Poland

**Keywords:** alcohol-related liver disease, cognitive impairment, liver transplantation, blood ammonia level, hepatic encephalopathy

## Abstract

Cognitive impairment (CI) in alcohol-related liver cirrhosis (ALD) is often underestimated, primarily attributed to hepatic encephalopathy (HE), despite evidence suggesting that deficits may persist after liver transplantation (LT). This study assessed CI both before and after LT through a structured psychiatric evaluation. A total of 101 ALD patients listed for LT were assessed; 61 underwent transplantation. Three patients died pre-LT, and six post-LT, leaving 55 for longitudinal cognitive evaluation. The Addenbrooke’s Cognitive Examination III (ACE III) was administered at LT listing and 7.1 months post-LT. Pre-LT CI was prevalent, with 86% scoring below the ACE III threshold. Mild cognitive impairment (MCI) was observed in 33%, and 52% had a high probability of dementia. Post-LT, ACE III scores improved (Δ +7.07 ± 8.47, P < 0.01), with the greatest gains in memory (+1.46, P = 0.01) and verbal fluency (+1.43, P = 0.02), while attention remained largely unchanged. Despite overall cognitive recovery, persistent deficits were observed, particularly in executive function and fluency. LT improves cognition, but persistent deficits suggest CI in ALD is not entirely reversible. These findings underscore the need for targeted cognitive interventions before and after LT.

## Graphical Abstract

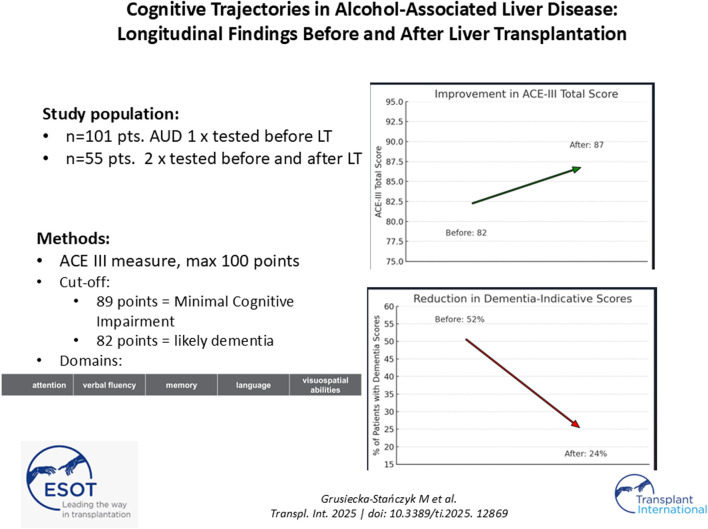

## Introduction

Alcohol is the most widely abused psychoactive substance globally, with high-risk drinking reported in up to 30% of Western populations, contributing substantially to morbidity and mortality [1]. Alcohol use disorder (AUD), as defined by the Diagnostic and Statistical Manual of Mental Disorders, 5th edition (DSM-V), encompasses a spectrum of maladaptive drinking behaviors that result in clinically significant physical, psychological, or social dysfunction [2]. Excessive alcohol intake is a well-established cause of liver cirrhosis, which remains a leading indication for liver transplantation (LT), particularly in cases of severe alcoholic hepatitis and end-stage liver disease (ESLD) [3, 4].

Cirrhosis-related neurocognitive decline is commonly attributed to hepatic encephalopathy (HE), a complication of advanced liver dysfunction associated with hyperammonemia and disruption of the liver-brain axis [5, 6]. While blood ammonia levels are a recognized biomarker of HE, they primarily have a high negative predictive value and do not reliably correlate with cognitive impairment (CI) [5, 6]. Although HE is considered reversible after LT, studies indicate that some patients with overt HE pre-transplantation exhibit persistent or even worsening cognitive deficits post-LT, suggesting that CI may result from more complex and multifactorial mechanisms [7, 8].

In patients with AUD, cognitive deficits extend beyond HE. Chronic alcohol consumption leads to widespread and potentially irreversible neurotoxic effects, including oxidative stress, mitochondrial dysfunction, and neuroinflammation. Acetaldehyde, the primary metabolite of ethanol, induces cellular damage by forming protein adducts and generating reactive oxygen species, which impair mitochondrial DNA and neuronal integrity [9]. Concurrently, elevated levels of pro-inflammatory cytokines—such as TNF-α, IL-6, IL-1β, and MCP1—are known to disrupt neuroplasticity and contribute to structural and functional changes in key brain regions, including the hippocampus and prefrontal cortex [10, 11].

As a result, individuals with AUD are particularly susceptible to a broad spectrum of cognitive deficits, including impairments in executive function, attention, abstract reasoning, psychomotor speed, visuospatial skills, language, and both verbal and visual memory [12–14]. These deficits are often linked to alcohol-induced reductions in hippocampal white matter volume and damage to the prefrontal cortex—areas crucial for memory, decision-making, and behavioral regulation. Structural brain abnormalities, minimal and overt HE, chronic alcohol use, diabetes, and gut microbiome dysbiosis may further contribute to pre-transplant CI and could influence its persistence after LT [15, 16].

Despite these known associations, cognitive impairment in patients with alcohol-related liver cirrhosis awaiting LT remains under-investigated. Existing studies on CI in LT recipients with cirrhosis of mixed etiologies report a post-transplant prevalence of CI ranging from 0% to 36% [17], but they are limited by methodological inconsistencies, variable definitions of HE and CI, and heterogeneous patient populations. Additionally, most prior research originates from neurology or psychiatry domains, often employing diverse and non-standardized diagnostic tools, which complicates comparisons and limits clinical applicability [18–20].

To address these limitations, the present pilot study aimed to perform a structured, longitudinal evaluation of cognitive function in a homogeneous cohort of patients with AUD-related ESLD. By assessing cognition both at the time of listing and after liver transplantation in a single-center setting, this study seeks to provide new insight into the nature, evolution, and clinical relevance of cognitive impairment in this vulnerable patient population.

Given the limited availability of long-term, prospective data on the trajectory of cognitive impairment (CI) in patients before and after liver transplantation (LT) - particularly in clinically homogeneous populations - two primary research objectives were formulated:

1. To conduct a quantitative and qualitative assessment of cognitive impairment prior to liver transplantation in patients with alcohol-related liver disease (ALD) qualified for transplantation within a single transplant center.
2. To analyze changes in cognitive function following liver transplantation, with particular emphasis on the dynamics and potential improvement of cognitive performance in this specific patient population.

## Patients and Methods

Between November 2022 and January 2024, a total of 101 consecutive adult patients with alcohol use disorder (AUD) were enrolled in the study (78% male; mean age: 53 ± 11 years; mean MELD score: 16 ± 7), all of whom were evaluated as potential candidates for liver transplantation (LT). Among them, 17% had hepatocellular carcinoma (HCC), while the remaining 83% were assessed for LT due to chronic liver failure.

Exclusion criteria included regular use of sedative medications, severe overt hepatic encephalopathy, and psychiatric or neurodegenerative disorders precluding cognitive assessment. No patients with acute alcoholic hepatitis were included in the study.

Plasma ammonia concentration was measured using an enzymatic method with glutamate dehydrogenase (GLDH) on the Dimension EXL analyzer (Siemens Healthineers, Forchheim, Germany), with a reference range of 19–55 μg/dL.

**FIGURE 1:**
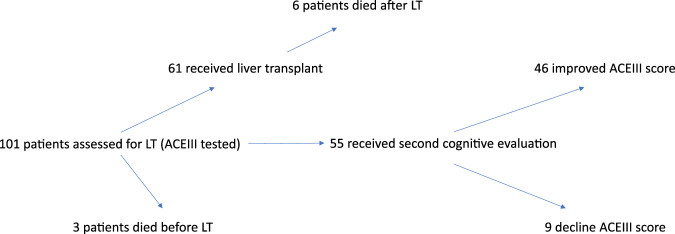
Flow chart of patient inclusion, liver transplantation outcomes, and cognitive assessment results before and after transplantation.

Of the 101 patients who underwent baseline cognitive assessment, 61 successfully received liver transplants. Three patients died before LT and an additional six died postoperatively. Ultimately, 55 individuals (including 14 women, 25%) underwent repeated cognitive evaluation (Figure 1). The mean age in this subgroup was 56 years.

All patients were routinely assessed during hospitalization by a psychiatrist dedicated to the transplant program, as part of the standard pre-transplant evaluation protocol. This included a comprehensive psychiatric consultation and administration of the Addenbrooke’s Cognitive Examination III (ACE-III). In patients who completed both assessments, psychiatric evaluation was conducted at listing for liver transplantation and again at an average of 7.1 months post-transplantation (SD = 1.45; range: 6–11 months; median: 7 months), ensuring methodological consistency and enabling reliable longitudinal analysis.

### Cognitive Functions Assessment

The Addenbrooke Cognitive Test III (ACE III) with a cut-off of 89 points for Mild Cognitive Impairment (MCI), and <82 points for a high probability of dementia was used to evaluate cognitive functions. The ACE III test covers five main domains of cognition, i.e., attention, verbal fluency, memory, language and visuospatial abilities, and is widely used, due to its specificity and sensitivity as well as simplicity and feasibility for administration, not only for physicians. The Polish version is available free of charge [21]. The ACE III was previously validated using standard neuropsychological tests [22]. Beside this, the center already has own experience in using this diagnostic tool, in the form of projects already published [23, 24].

### Statistical Analyses

Statistical analyses were performed using SPSS (SPSS Statistics, version 28.0. IBM Corp., USA). Continuous variables are shown as mean ± standard deviation (SD) and categorical variables are expressed as absolute and relative (in per cent) frequencies. The Kolmogorov‐Smirnov test was applied to determine whether continuous variables were normally distributed. The Wilcoxon Mann‐Whitney U test or Student´s t‐test were used for analyzing continuous variables. Chi^2^ test and Fisher´s exact test were used for group comparisons of categorical variables. The correlations between ACE III results and clinical variables were evaluated by Spearman’s rank correlation coefficient. The associations between ACE III result suggesting probability of dementia (ACE III <82 points) and clinical data were assessed by univariate and multivariate logistic regression analyses. Statistical procedures were performed two‐sided and p‐values <0.05 reflected to be statistically significant.

### Ethics

Appropriate informed consent was obtained from each patient included in the study. The study protocol was approved by the Bioethics Committee of the Medical University of Warsaw (approval number KB/81/2022) and conforms with the ethical guidelines of the 1975 Declaration of Helsinki (6th revision, 2008).

## Results

### Preliminary Assessment of Cognitive Function

At the initial stage of the study, cognitive function was assessed in 101 individuals. Overall, cognitive impairment, as evaluated using the Addenbrooke’s Cognitive Examination III (ACE-III), was observed in 86% of participants: 33% met the criteria for mild cognitive impairment (MCI), while 52% met the criteria indicating a high likelihood of dementia.

The mean total ACE-III score was 78.32 ± 11.99, ranging from 50 to 96, with a median of 79.0 (interquartile range [IQR]: 70.0–87.0).

Among the five cognitive domains assessed, the greatest deficits were observed in verbal fluency, followed by visuospatial abilities. The mean scores for attention, memory, language, and visuospatial skills were 16.11, 17.34, 22.43, and 11.84 points, respectively, with median scores of 17.0, 18.0, 23.0, and 12.0. Details are presented in Table 1. As variables followed non-normal distributions (as per Kolmogorov–Smirnov test), both means (±SD) and medians (IQR) are reported for all ACE III subdomains to enhance interpretability.

**TABLE 1:** ACE-III cognitive performance among liver transplant candidates with ALD.

N = 101	Age	Child-Pugh class	MELD	ACEIII	Attention	Memory	Fluency	Langugage	Visuo-spatial abilities	Years of education
Mean ± SD	52.54 ± 10.52	8.28 ± 1.94	16.0 ± 6.56	78.32 ± 11.99	16.11 ± 1.96	17.34 ± 4.66	10.21 ± 2.48	22.43 ± 3.98	11.84 ± 3.29	12.35 ± 2.91
median	53.0	8.0	14.0	81.0	17.0	18.0	10.0	24.0	12.0	12.0
range	24–73	5–14	6–41	42–100	8–18	3–26	3–14	4–26	1–17	8–20
IQR	45–61	7–10	11–20	71–86	15–18	15–20	9–12	21–25	10–15	10–13

The mean venous blood ammonia concentration was 86 ± 57 μg/dL, with hyperammonemia (>55 μg/dL) observed in 45% of patients. As shown in Table 1, patients exhibiting signs of dementia had significantly shorter education duration (*P* < 0.001) and higher Child-Pugh scores (*P* = 0.04). However, no other clinical differences were identified—such as ammonia level or MELD score—when compared to patients with ACE-III scores >82.

The likelihood of dementia was significantly higher in patients with alcohol-related liver disease (ALD) alone compared to those with ALD and concomitant hepatocellular carcinoma (HCC) (P = 0.015). Among the 17 patients with ALD + HCC, 4 (23.5%) had ACE-III scores below 82, indicating a high probability of dementia. In contrast, 52% of patients in the ALD-only group fell below this threshold.

A total of ten deaths were recorded in the cohort, with a trend toward higher mortality among patients scoring <82 on the ACE-III (15%) versus those scoring ≥82 (4%), though this did not reach statistical significance (*P* = 0.066), as shown in Table 2. Moreover, 76 individuals (75%) were listed for liver transplantation, but no significant differences were observed in ACE-III total scores or individual cognitive domains based on transplant listing status.

**TABLE 2:** Clinical characteristics of the study group and subgroups with positive and negative screening results for suspected dementia based on the ACE-III test (i.e., <82 points).

	Entire cohort	ACE III positive for dementia suspicion	ACE III negative for dementia suspicion	P – value
N, %	101 (100%)	53 (52.5%)	48 (47.5%)	-
Age, years	52.5 ± 10.5	53.3 ± 10.0	51.7 ± 11.1	0.304
Females, n (%)	22 (21.8%)	11 (20.8%)	11 (22.9%)	0.814
MELD (points)	16.0 ± 6.6	16.9 ± 7.0	15.0 ± 6.0	0.114
Child-Pugh (points)	8.3 ± 1.9	8.7 ± 1.9	7.9 ± 2.0	0.044
Blood ammonia, ng/mL	85.5 ± 56.8	82.8 ± 45.8	88.6 ± 67.5	0.961
Years of education	12.4 ± 2.9	11.2 ± 2.2	13.7 ± 3.1	<0.001
ACE III, points	78.3 ± 12.0	70.1 ± 10.8	87.4 ± 4.2	<0.001
Death, n (%)	10 (10%)	8 (15%)	2 (4%)	0.066

Abbreviations: ACE-III, Addenbrooke’s Cognitive Examination III; MELD, Model for End-Stage Liver Disease score.

The mean total ACE-III score in the entire cohort was 78 ± 12 points, with the lowest scores observed in the domains of verbal fluency and visuospatial abilities. The ACE-III score was significantly correlated with years of education and the Child-Pugh score, as shown in Table 3.

**TABLE 3:** Correlations between variables and ACE-III total and domain scores, presented as rho values.

	Total ACE III	Attention ACEIII	Verbal fluency ACEIII	Memory ACEIII	Language ACE III	Visuospatial abilities ACEIII
Age	−0.190	−0.098	−0.262**	−0.136	−0.146	−0.129
Female	0.016	−0.022	0.008	−0.044	−0.030	0.118
Education period	0.390**	0.225*	0.077	0.417**	0.139	0.266**
Child-Pugh score	−0.262**	−0.320**	−0.253*	−0.121	−0.270**	−0.200*
MELD score	−0.161	−0.240*	−0.177	−0.098	−0.251*	−0.076
Blood ammonia level	−0.075	−0.063	−0.091	−0.063	−0.056	−0.132
Total ACE III	1	0.522**	0.714**	0.717**	0.601**	0.746**

Abbreviations: ACE-III, Addenbrooke’s Cognitive Examination III; MELD, Model for End-Stage Liver Disease score. Rho values were calculated using Spearman’s rank correlation coefficient, and p values <0.05 were considered statistically significant.

* –*p* < 0.05; ** –*p* < 0.001.

Notably, the overall MELD score was not associated with the total ACE-III score; however, it showed a significant correlation with the attention and language subdomains of the ACE-III (Table 3). Additionally, age was negatively correlated with the verbal fluency subscale and showed a negative trend in relation to the overall ACE-III score (Table 3).

Multivariable analysis demonstrated that both the Child-Pugh score (OR 1.51; 95% CI: 1.14–2.00) and years of education (OR 0.65; 95% CI: 0.53–0.79) were independently associated with ACE-III scores below 82 points, indicating a high likelihood of dementia (Table 4). In contrast, age, sex, blood ammonia level, presence of hyperammonemia, and MELD score showed no significant association with ACE-III scores <82.

**TABLE 4:** Logistic regression analysis for ACE-III scores <82 in patients with alcohol-related liver cirrhosis.

	Univeriable		Multivariable	
	*P* value	OR (95% CI)	*P* value	OR (95% CI)
Years of education	<0.001	0.70 (0.59–0.84)	<0.001	0.65 (0.53–0.79)
Child-Pugh score	0.039	1.25 (1.01–1.55)	0.004	1.51 (1.14–2.00)
MELD score	0.133	1.05 (0.99–1.12)	-	
Age	0.413	1.02 (0.98–1.05)	-	
Blood ammonia	0.612	1.00 (0.99–1.01)	-	
Hiperammonemia	0.732	0.87 (0.40–1.92)	-	
Females	0.793	0.88 (0.34–2.27)	-	

Abbreviations: ACE-III, Addenbrooke’s Cognitive Examination III; MELD, Model for End-Stage Liver Disease score; OR, odds ratio.

### Results of Repeated Cognitive Assessment (N = 55 Patients)

#### Cognitive Function Before Liver Transplantation

Before transplantation, the mean ACE-III score was 80.62 ± 10.40, with a median of 82.0 (range: 51–97; IQR: 75.0–87.0). Among the assessed cognitive domains, the language domain was best preserved (mean: 23.19 ± 3.44), while the greatest deficits were observed in verbal fluency (mean: 10.21 ± 2.48). Memory and visuospatial abilities showed moderate performance (memory: 18.15 ± 4.16; visuospatial: 12.22 ± 3.15). The mean attention score was 16.38 ± 1.39.

The average number of years of education in this group was 12.19 ± 3.24.

Data are presented in Table 5.

**TABLE 5:** ACE-III results among liver transplant recipients with alcohol-related liver disease (ALD).

N = 55	Age	ACEIII 1	Attention 1	Memory 1	Fluency 1	Language 1	Visuo-spatial abilities 1	Year of education
Mean ±SD	53.19 ± 9.72	80.62 ± 10.40	16.38 ± 1.39	18.15 ± 4.16	10.21 ± 2.48	23.19 ± 3.44	12.22 ± 3.15	12.19 ± 3.24
median	53.0	82.0	17.0	19.0	10.0	24.0	13.0	12.0
range	32–74	51–97	12–18	8–25	3–14	10–26	3–16	8–20
IQR	46.0–60.0	75.0–87.0	16.0–18.0	16.0–22.0	9–12	21.0–25.0	10.0–14.0	10.0–14.0

#### Cognitive Function After Liver Transplantation (n = 55 Patients)

An overall improvement in cognitive function was observed following liver transplantation. The mean ACE-III score increased to 87.0 ± 6.7 points, with a median of 86.0 (range: 73–98; interquartile range [IQR]: 83.0–91.0).

The most notable improvements were seen in memory (mean: 20.83 ± 3.88; median: 21.0) and verbal fluency (mean: 12.08 ± 1.56; median: 12.0). Improvement was also evident in attention (mean: 17.0 ± 1.18), language (mean: 23.85 ± 2.23), and visuospatial abilities (mean: 13.93 ± 2.14). Data are presented in Table 6.

**TABLE 6:** Second ACE-III assessment in liver transplant recipients with alcohol-related liver disease (ALD).

N = 55	ACEIII 2	Attention 2	Memory 2	Fluency 2	Language 2	Visuo-spatial abilities 2	Time form LT (months)
Mean ±SD	87.0 ± 6.7	17.0 ± 1.18	20.83 ± 3.88	12.08 ± 1.56	23.85 ± 2.23	13.93 ± 2.14	7.13 ± 1.45
median	86.0	17.0	21.0	12.0	25.0	14.0	7.0
range	73–98	13–18	10–25	9–14	15–26	7–16	6–11
IQR	83.0–91.0	16.0–18.0	18.0–24.0	11.0–13.0	22.0–25.0	13.0–15.0	6.0–8.0

#### Changes in Cognitive Function Following Liver Transplantation (Δ Scores)

Following liver transplantation, patients exhibited a mean increase of 7.07 ± 8.47 points in total ACE-III scores (median: +5.0; range: –10 to 34; interquartile range [IQR]: 2.0–9.0). The most pronounced improvements were observed in the following cognitive domains:

• Memory: +1.46 ± 2.91 (median: +1.0; range: –7 to 10; IQR: 0.0–3.0)
• Verbal Fluency: +1.43 ± 1.89 (median: +2.0; range: –3 to 6; IQR: 0.0–2.0)
• Visuospatial Abilities: +1.52 ± 2.57 (median: +2.0; range: –5 to 7; IQR: 0.0–3.0)
• Attention: +0.71 ± 1.23 (median: +1.0; range: –3 to 4; IQR: 0.0–1.0)
• Language: +0.32 ± 2.54 (median: 0.0; range: –4 to 14; IQR: –1.0–1.0)

A Wilcoxon signed-rank test confirmed that the observed post-transplant improvement in total ACE-III scores was statistically significant (*Z* ≈ 420.0, *p* < 0.0001), indicating that the changes were unlikely to have occurred by chance. Moreover, statistically significant gains were also observed across most individual subdomains (*all p* < 0.01), further supporting the robustness of the cognitive recovery pattern.

**FIGURE 2:**
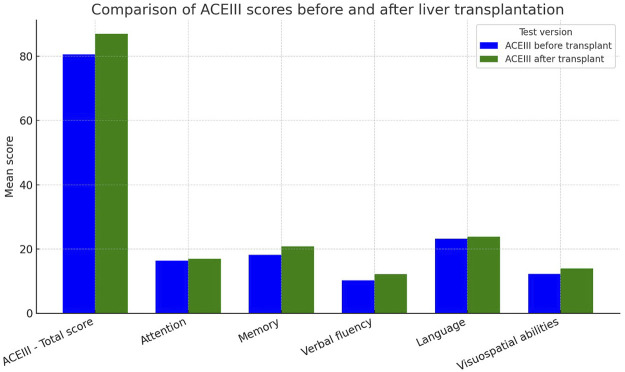
ACE-III scores before and after liver transplantation in recipients with alcohol-related end-stage liver disease.

Among the 55 patients assessed, 42 (76%) demonstrated cognitive improvement, 9 (16%) experienced decline, and 4 (8%) remained stable. This distribution was also statistically significant (Wilcoxon signed-rank test, *p* < 0.01) and is depicted in Figure 2.

**Table:** 

Test	Z/W-statistic	p-value
Wilcoxon	≈ **420.0**	**< 0.0001**

#### Cognitive Decline After Liver Transplantation

Although the overall trajectory pointed toward cognitive improvement, a decline in total ACE-III scores was observed in 16.4% of patients (9 out of 55). Specific cognitive domains most frequently affected by post-transplant deterioration included:

• Language–decline in 29.1% of patients (16/55)
• Memory–decline in 20.0% of patients (11/55)
• Attention–decline in 16.4% of patients (9/55)
• Verbal Fluency–decline in 14.5% of patients (8/55)
• Visuospatial Abilities–decline in 10.9% of patients (6/55)

**FIGURE 3:**
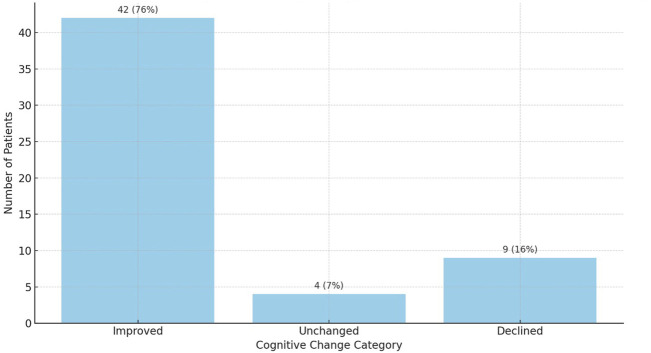
Distribution of cognitive change categories after liver transplantation based on ACE-III scores (n = 55). Cognitive improvement was observed in 42 patients (76%), decline in 12 (22%), and no change in 1 patient (2%).

The overall distribution of cognitive change categories is illustrated in Figure 3, showing that the majority of patients (n = 42; 76%) demonstrated post-transplant cognitive improvement, while 16% (n = 9) experienced decline and 7% (n = 4) remained unchanged (Figure 3).

#### Exploratory Comparison: Improved vs. Declined Cognitive Trajectory

To further explore factors associated with cognitive outcomes following liver transplantation, we performed an exploratory subgroup analysis comparing patients who demonstrated cognitive improvement with those who experienced decline, based on changes in total ACE-III scores 6 months post-transplant. On average, patients in the improved group were younger (55.4 ± 8.8 vs. 59.3 ± 7.4 years) and had more years of education (12.7 ± 2.9 vs. 11.4 ± 3.1 years). The difference in educational attainment between the groups reached statistical significance (Mann–Whitney *U*, *p* = 0.045), while the difference in age did not (*p* = 0.165).

In a broader comparison including patients with no change in cognitive performance (n = 4), Kruskal–Wallis testing revealed a trend toward significance for years of education (*p* = 0.072), with age differences remaining non-significant (*p* = 0.148). These findings suggest that educational background may play a role in cognitive recovery after liver transplantation, although results should be interpreted cautiously due to the limited sample size. A summary of this exploratory analysis is presented in Table 7.

**TABLE 7:** Comparison of patients with improved vs. declined cognitive performance post-transplant.

Group	N	Age (mean ± SD)	Education years (mean ± SD)
Improved	12	55.4 ± 8.8	12.7 ± 2.9
Declined	9	59.3 ± 7.4	11.4 ± 3.1
No change	4	52.0	10.5
p-value (Kruskal-Wallis)		0.148	0.072
p-value (Mann–Whitney U)		0.165	0.045

Due to non-normal distribution and small sample sizes, non-parametric tests were used: Kruskal–Wallis for three-group comparisons and Mann–Whitney U for pairwise testing.

## Discussion

This study provides a comprehensive evaluation of cognitive function at the time of liver transplant listing and in the early post-transplant period in a homogeneous cohort of patients with alcohol use disorder (AUD)-related liver cirrhosis. Cognitive impairment (CI) is increasingly recognized in patients with end-stage liver disease (ESLD), but available data remain heterogeneous, encompassing mixed etiologies, various diagnostic tools, and differing disease severity. Thus, a critical gap persists in understanding the true prevalence and profile of CI in well-defined liver transplant candidate populations.

To the best of our knowledge, this is the first study to examine cognitive performance both before and after liver transplantation (LT) in a cohort exclusively composed of patients with AUD-related ESLD. It delivers new insights into a crucial yet underexplored dimension of peri-transplant care. A recent review by Siddiqui et al. [17] included 24 studies with a median of 30 patients per study and follow-up ranging from 1 month to 1.8 years post-LT. The prevalence of CI varied from 4% to 36% within the first 8 months post-transplant and from 0% to 16% thereafter. Due to methodological variability, CI was grouped into six cognitive domains: attention, executive functions, working memory, long-term memory, language, and visuospatial abilities. However, no prior study has provided precise pre-LT CI data specifically in AUD candidates.

Our single-center analysis demonstrated a strikingly high prevalence of significant CI suggestive of dementia, with only 14% of patients scoring within the normal range on the ACE-III. The lowest performance was noted in verbal fluency and visuospatial domains, echoing findings by Lee et al. [25] and Sorrell et al. [26], who reported deficits in memory, visuospatial construction, attention, and immediate memory in patients with alcohol-related cirrhosis. Prior work by our group also showed significantly higher ACE-III scores and lower CI prevalence in patients with end-stage kidney disease (ESKD) compared to those with ESLD, with memory and visuospatial function being particularly impaired in the ESLD cohort [27].

Alcohol-induced brain damage is influenced by the quantity, age of onset, and duration of drinking, along with age, education, genetics, and prenatal exposure [14, 20, 28]. Stavro et al. [29] and Crowe et al. [28] described diffuse cognitive deficits in AUD, consistent with our cohort’s mean ACE-III score of 78, with dysfunction across all domains. Verbal fluency and visuospatial impairments were particularly prominent, aligning with findings from Crowe et al. [28] and others [30–34]. Post-transplant studies by Campagna et al. [15] and Siddiqui et al. [17] also reported persistent deficits in attention, executive function, memory, and visuospatial processing.

Although the pathophysiological mechanisms linking ESLD and CI remain complex, factors such as neuroinflammation, hippocampal atrophy, and oxidative damage to the prefrontal cortex have been implicated [13, 35, 36]. MRI studies have shown reduced hippocampal volume in AUD patients compared to healthy controls [37]. Our data revealed a significant negative correlation between age and verbal fluency, with a trend toward lower total ACE-III scores in older patients. This supports findings by Campagna et al. [15], who suggested a critical vulnerability window at ages 50–60, possibly reflecting cumulative alcohol-related neurotoxicity.

Educational attainment emerged as a protective factor against CI, with ACE-III scores positively correlated with years of education, in line with Schneeweis et al. [38]. Conversely, a higher Child-Pugh score was associated with poorer cognitive outcomes and an increased likelihood of dementia-range ACE-III results. The MELD score did not correlate with total ACE-III performance but did show associations with attention and language subdomains. This divergence may relate to MELD’s omission of nutritional parameters like serum albumin. Chronic malnutrition and sarcopenia, common in advanced liver disease, are known contributors to cognitive dysfunction [39–41].

Our findings align with reports showing greater post-LT CI prevalence in patients with pre-transplant MELD scores of 22–26 (21%–36%) compared to those with scores of 11–19 (8%–13%) [16, 17]. These trends highlight the multifactorial burden of CI in advanced liver disease.

In our cohort of 55 patients who completed both pre- and post-transplant cognitive evaluations, we observed a significant overall improvement in ACE-III scores, with the most notable gains in memory, verbal fluency, and visuospatial domains. Language function showed only modest improvement and remained one of the most frequently impaired areas. These findings align with prior reports suggesting that LT may reverse cognitive deficits, particularly those associated with hepatic encephalopathy and systemic inflammation [15, 17]. The Wilcoxon signed-rank test confirmed the statistical significance of this improvement (p < 0.0001), further supporting the role of LT in promoting cognitive recovery in the majority of patients (Figure 2).

However, as illustrated in Figure 3, recovery was not universal - approximately 16% of patients experienced cognitive decline, most often in language and memory domains, while 8% remained unchanged. This variation highlights the need for long-term neurocognitive surveillance and for identifying risk factors associated with suboptimal outcomes.

Preliminary findings (Table 7) suggest that younger age and higher educational attainment may offer some protection against cognitive decline following liver transplantation. Given the high prevalence and clinical consequences of cognitive impairment in patients with alcohol-related end-stage liver disease, routine cognitive assessment during pre-transplant evaluation appears warranted. Early detection of individuals at increased risk could enable personalized cognitive support strategies and potentially enhance long-term clinical outcomes. CI has been shown to negatively impact treatment adherence, decision-making capacity, and overall prognosis in transplant recipients [42].

The association between higher educational attainment and cognitive improvement post-transplant is consistent with the cognitive reserve hypothesis, which posits that individuals with greater lifelong cognitive engagement - often reflected by formal education - may be more resilient to the effects of brain injury, including those related to hepatic encephalopathy. Although the current sample size is limited, the statistically significant difference in education between improved and declined patients lends further support to this concept.

Notably, age did not significantly differentiate outcome groups, suggesting that within this cohort, cognitive reserve may have been a more relevant determinant of cognitive recovery than chronological age.

Future studies should aim to incorporate neuroimaging and biomarkers to elucidate the mechanisms underlying CI. Clinical trials of cognitive training and pharmacologic interventions in this population are also needed to guide individualized care strategies.

### Limitations

This study has several limitations. First, data on the duration and quantity of alcohol consumption were not collected. However, all patients had abstained from alcohol for at least 6 months, verified by psychiatric assessment. Second, DSM-5 criteria were not applied to diagnose major or minor neurocognitive disorders. Nonetheless, ACE-III testing was performed by a psychiatrist during standard pre-transplant evaluation, following exclusion of major neurological conditions via MRI. Third, minimal hepatic encephalopathy was not assessed using tools such as the MMSE due to the pilot nature of the study.

Additionally, comparing our findings with previous research is challenging due to variability in study populations, methodologies, and sample sizes. Despite these limitations, our study provides important data from a relatively large, etiologically homogeneous cohort of AUD-related ESLD patients, showing a disturbingly high prevalence of CI. Routine cognitive screening may facilitate earlier interventions and improved clinical management.

## Conclusion

This study demonstrates a concerning prevalence of severe cognitive impairment, potentially indicative of dementia, in patients with alcohol-related ESLD both before and after liver transplantation. Although most patients showed post-transplant improvement, persistent deficits in memory and language highlight the need for ongoing monitoring. Our findings support routine cognitive screening in this population and underscore the importance of further research into predictive markers and therapeutic interventions aimed at preserving and enhancing cognitive function after LT.

## Data Availability

The raw data supporting the conclusions of this article will be made available by the authors, without undue reservation.
